# Association Between Heparin Dose, Body Mass Index, and Stroke Risk in Patients Undergoing TAVR

**DOI:** 10.3390/jcm15031201

**Published:** 2026-02-03

**Authors:** Ziad Arow, Juri Iwata, Akiko Masumoto, Arthur Clement, Laurent Lepage, Laurent Bonfils, Rawia Hussein-Aro, Abid Assali, Nicolas Dumonteil, Didier Tchetche, Chiara De Biase

**Affiliations:** 1 Groupe Cardio Vasculaire Interventionnel, Clinique Pasteur, 45 Avenue de Lombez, 31076 Toulouse, France; ziad.arow@gmail.com (Z.A.); juriandbbpomecr@gmail.com (J.I.); akikomasu@gmail.com (A.M.); arthur.clementvt@gmail.com (A.C.); laulepage@yahoo.fr (L.L.); lbonfils@clinique-pasteur.com (L.B.); ndumonteil@clinique-pasteur.com (N.D.); dtchetche@clinique-pasteur.com (D.T.); 2 Cardiology Department, Meir Medical Center, Sackler Faculty of Medicine, Tel Aviv University, Tel Aviv 6139001, Israel; aassali@clalit.org.il; 3 Hillel Yaffe Medical Center, Technion—Israel Institute of Technology, Haifa 3200003, Israel; rawiahu@gmail.com

**Keywords:** transcatheter aortic valve replacement (TAVR), unfractionated heparin (UFH), activated clotting times (ACT), peri-procedural stroke, body mass index (BMI)

## Abstract

**Background:** Unfractionated heparin (UFH) is routinely administered during transcatheter aortic valve replacement (TAVR) to prevent thromboembolic complications. However, there are no clear evidence-based guidelines defining optimal heparin dosing or target activated clotting time (ACT) values. This study aimed to evaluate the association between intraprocedural UFH dosing, ACT values, and peri-procedural stroke risk in the overall population of patients undergoing TAVR, with a prespecified stratified analysis according to body mass index (BMI ≥ 30 vs. <30 kg/m^2^). **Methods:** This analysis enrolled consecutive individuals with severe aortic stenosis (AS) who were treated with TAVR using either balloon-expandable or self-expanding valves. The primary outcome was the occurrence of stroke during the periprocedural period in the overall population and according to BMI (<30 vs. ≥30 kg/m^2^). Secondary endpoints included periprocedural parameters, clinical outcomes (in-hospital and 1-year mortality), and safety outcomes. Subgroup analysis was performed to assess stroke risk according to ACT values. Patients with atrial fibrillation or receiving chronic oral anticoagulation were excluded. **Results:** A total of 1045 patients underwent TAVR between 2022 and 2024, including 827 with BMI < 30 and 218 with BMI ≥ 30. The study population had a mean age of 82 ± 6 years, and 56% of patients were male. In the overall study population, the mean heparin dose was 47 U/kg and the mean ACT value was 218 s. Patients with lower BMI received higher heparin doses (50 vs. 40 U/kg, *p* < 0.01) and had higher ACT values (221 vs. 208 s, *p* < 0.01). Protamine use was low and similar between groups. Periprocedural stroke rates were low overall (1.1%) and comparable between study groups (1.2% vs. 0.9%, *p* = 0.71). One-year mortality was also similar (3% vs. 4%, *p* = 0.53), with no significant differences in other safety outcomes. Subgroup analysis by ACT (≤250 vs. >250 s) showed no difference in stroke rates (1% vs. 1.5%, *p* = 0.60). **Conclusions:** In this single-center cohort, differences in heparin dosing and ACT values were not associated with differences in peri-procedural stroke or overall procedural outcomes. However, given the low number of stroke events, these findings should be interpreted cautiously. Prospective randomized studies are needed to define optimal anticoagulation strategies during TAVR.

## 1. Introduction

Severe aortic stenosis (AS) is a progressive and life-threatening disease if left untreated [1,2,3,4]. Transcatheter aortic valve replacement (TAVR) has become an established, life-prolonging treatment for individuals with severe aortic stenosis [3,4,5,6,7]. During TAVR, unfractionated heparin (UFH) is routinely administered intravenously to prevent thromboembolic complications associated with large-bore arterial access, catheter manipulation, and valve deployment. Unlike percutaneous coronary intervention (PCI) or diagnostic coronary angiography, where clear evidence-based guidelines exist for heparin dosing and target activated clotting times (ACT) [8], current recommendations for anticoagulation during TAVR are largely expert consensus and not supported by high-quality evidence [9]. Previous recommendations have suggested weight-adjusted dosing of UFH with a target ACT greater than 300 s [10]. However, other studies and recent recommendations have reported that an ACT target range of 250 to 300 s during TAVR procedures might be considered [9,11]. Current evidence supports the use of ACT-guided heparin administration, which has been associated with favourable safety outcomes [12]. Consequently, the heparin dose, ACT targets, and the use of reversal agents such as protamine vary considerably among operators and centers, resulting in marked heterogeneity in clinical practice. The same uncertainty applies to dosing adjustments in obese patients, where higher doses of heparin are often administered empirically, yet no specific guidelines exist to direct these practices.

Peri-procedural stroke is a serious complication of TAVR. Although stroke rates after TAVR are generally lower than those observed after surgical aortic valve replacement (SAVR) [13], it remains clinically significant and is associated with a two- to nine-fold increase in mortality [14]. These events underscore the importance of optimizing procedural strategies, including anticoagulation management, to minimize thromboembolic risk while maintaining safety.

The objective of the present study was to evaluate the association between intraprocedural UFH dosing, ACT values, and peri-procedural stroke risk in the overall population of patients undergoing TAVR, and to compare the heparin dose administered, corresponding ACT values, and peri-procedural stroke risk between obese (BMI ≥ 30 kg/m^2^) and non-obese patients undergoing TAVR.

## 2. Methods

This retrospective single-center clinical registry study was performed at Clinique Pasteur in Toulouse, France. The dataset included all consecutive patients who underwent TAVR for severe AS between 2022 and 2024 using either balloon-expandable valves (Sapien 3, Sapien 3 Ultra, Edwards Lifesciences, Irvine, CA, USA; Colibri, Colibri Heart Valve, Broomfield, CO, USA; and MyVal, Meril Life Sciences, Vapi, Gujarat, India) or self-expandable valves (Evolut R, Evolut Pro, Evolut Pro+, Medtronic, Minneapolis, MN, USA; Navitor, Abbott, Abbott Park, IL, USA; and ACURATE neo, Boston Scientific, Marlborough, MA, USA). Eligibility for TAVR was assessed according to the European Society of Cardiology guidelines for valvular heart disease [3] and final treatment decisions were made by a multidisciplinary Heart Team. Patients receiving ongoing oral anticoagulation, those with history of atrial fibrillation, and individuals undergoing valve in valve or Transcatheter Aortic Valve (TAV) in TAV procedures were excluded from the current analysis. The study included patients undergoing TAVR at Clinique Pasteur whose clinical and procedural data were recorded in the national FRANCE TAVI registry. This study is reported in accordance with the Strengthening the Reporting of Observational Studies in Epidemiology (STROBE) statement, and the completed STROBE checklist is provided in the Supplementary Material (Supplementary Table S1). The study population had a mean age of 82 ± 6 years, and 56% were male. Baseline demographic, clinical, and echocardiographic variables were collected for all included patients, and baseline characteristics of the overall cohort and according to BMI group are summarized in Table 1.

The primary endpoint was the incidence of peri-procedural stroke in the overall population of patients undergoing TAVR, with a prespecified stratified analysis according to body mass index (BMI ≥ 30 vs. <30 kg/m^2^). Stroke events were evaluated during in-hospital follow-up and were limited to events occurring before discharge. Stroke events were assessed and first identified based on clinical suspicion by the treating cardiologist before hospital discharge, with neurological assessment performed only after the initial clinical evaluation and suspicion of stroke by the treating physician. They were subsequently confirmed by cerebral imaging, consisting of computed tomography (CT) and/or magnetic resonance imaging (MRI), and formal neurologist evaluation. Formal stroke severity scales, including the NIH Stroke Scale (NIHSS) and modified Rankin Scale (mRS), were not systematically recorded in the registry. Secondary endpoints included peri-procedural data (including procedure duration, heparin dose [U/kg], activated clotting time [ACT, seconds], and protamine administration according to BMI group). In routine practice at our center, UFH was administered as an initial fixed bolus according to BMI category (typically 3000 IU in patients with BMI < 30 kg/m^2^ and 5000 IU in patients with BMI ≥ 30 kg/m^2^). Additional UFH boluses were given at the operator’s discretion based on ACT results and procedural duration. ACT was first measured approximately 15 min after the initial UFH bolus and was rechecked if the procedure was prolonged or if initial ACT values were considered subtherapeutic. In general, ACT values around or below 200 s were considered subtherapeutic and could prompt additional UFH administration at the operator’s discretion. Total UFH dose (U/kg) reflects the sum of the initial and any additional boluses administered during the procedure.

Other secondary clinical outcomes included in-hospital and 1-year mortality, permanent pacemaker (PPM) implantation, and Safety outcomes (major vascular complications, life-threatening bleeding, major bleeding, coronary obstruction occurring during the procedure and peri-procedural myocardial infarction (MI)). In addition, a subgroup analysis was performed to assess stroke risk according to ACT values > 250 s versus ≤ 250 s. Clinical and procedural outcomes, including device success, bleeding, and vascular complications, were defined according to the Valve Academic Research Consortium-2 (VARC-2) criteria.

## 3. Statistical Analysis

Categorical and binary variables are expressed as frequencies and percentages, and group comparisons were performed using the Pearson chi-square test or Fisher’s exact test when appropriate.

The Kruskal–Wallis test was applied to evaluate differences in the distribution of continuous variables. Variables demonstrating a normal distribution are presented as mean ± standard deviation and were compared using an unpaired, two-tailed Student’s *t*-test. Continuous variables that did not follow a normal distribution are expressed as median with interquartile range and were analyzed using the Mann–Whitney U test.

Subgroup analysis was performed to assess stroke risk according to ACT values. Device success was evaluated as a key procedural endpoint because anticoagulation intensity may influence intra-procedural thrombotic complications, procedural performance, and the need for additional maneuvers, all of which are captured by VARC-defined device success. In addition, given the low number of stroke events, multivariable modeling for stroke was not feasible, whereas device success occurred more frequently and allowed exploratory adjusted analyses. Univariable and multivariable logistic regression models were constructed to determine predictors of device success. A backward stepwise selection approach was applied, incorporating variables that showed a *p*-value < 0.20 in the univariable analysis. All statistical tests were two-tailed, and a *p*-value below 0.05 was considered indicative of statistical significance. Data analysis was performed using SPSS software, version 29.0.2.0 (IBM Corp., Armonk, NY, USA).

## 4. Results

### 4.1. Baseline Characteristics

The analysis comprised 1045 patients who underwent TAVR, comprising 827 patients with a BMI < 30 and 218 patients with a BMI ≥ 30. In the overall study population, the mean age was 82 years and 56% of patients were male. The mean BMI was 27 kg/m^2^, with mean BMI values of 25 kg/m^2^ in the BMI < 30 group and 34 kg/m^2^ in the BMI ≥ 30 group. Baseline characteristics are summarized in Table 1 and Supplementary Table S2. Both study groups exhibited high rates of cardiovascular comorbidities. Patients in the higher BMI group had significantly higher rates of hypertension (87% vs. 72%, *p* < 0.001) and dyslipidemia (34% vs. 20%, *p* < 0.001), whereas a history of prior cerebrovascular accident or transient ischemic attack was more frequent in the lower BMI group (8% vs. 3%, *p* = 0.021). The aortic valve area (AVA) was slightly smaller in patients with lower BMI (0.79 cm^2^ vs. 0.82 cm^2^, *p* = 0.017), while mean transvalvular gradients were similar between groups (49 mmHg vs. 50 mmHg, *p* = 0.155). The EuroscoreII was modestly higher in the BMI < 30 group (median 2.45 vs. 2.32, *p* = 0.022). There were no significant differences in the prevalence of coronary artery disease between the groups (41% vs. 38%, *p* = 0.401). The rate of concomitant pre-TAVR PCI was similar as well, occurring in 22% of patients in the BMI ≥ 30 group versus 25% in the BMI < 30 group (*p* = 0.338). Overall, 81% of patients were treated with aspirin, with no statistically significant difference between the groups (80% vs. 82%, *p* = 0.534).

### 4.2. Periprocedural Data

**Figure 1:**
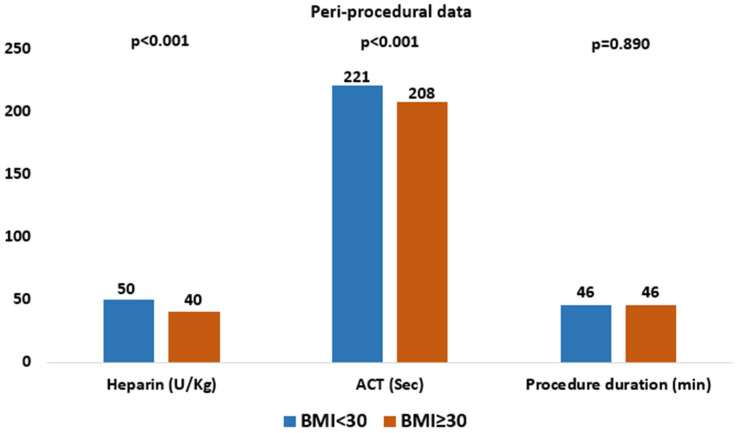
Peri-procedural data.

Periprocedural characteristics are summarized in Table 2 and Figure 1. In the overall study population (n = 1045), the majority of patients received self-expanding valves (64%), with a mean procedure duration of 46 min, a mean contrast volume of 82 mL, a mean heparin dose of 47 U/kg, and a mean ACT value of 218 s. The overall median ACT was 212 s (IQR 189–240). ACT values ≥ 250 s were achieved in 19% of patients, and ≥300 s in 7%. Compared with the high BMI group, patients in the low BMI group received a higher mean heparin dose (50 U/kg vs. 40 U/kg, *p* < 0.001) and had higher mean ACT values (221 s vs. 208 s, *p* < 0.001).

Protamine was not systematically administered to neutralize UFH, its use was low in the overall cohort (1.9%) and did not differ significantly between the study groups (2% vs. 1.3%, *p* = 0.515). There were no statistically significant differences between groups in the rates of pre-dilatation (24% vs. 21%, *p* = 0.319), post-dilatation (18% vs. 20%, *p* = 0.413), valve repositioning (34% vs. 33%, *p* = 0.762), or the need to implant a second valve during the procedure (1% vs. 0%, *p* = 0.217).

### 4.3. Procedural and Clinical Outcomes

**Figure 2:**
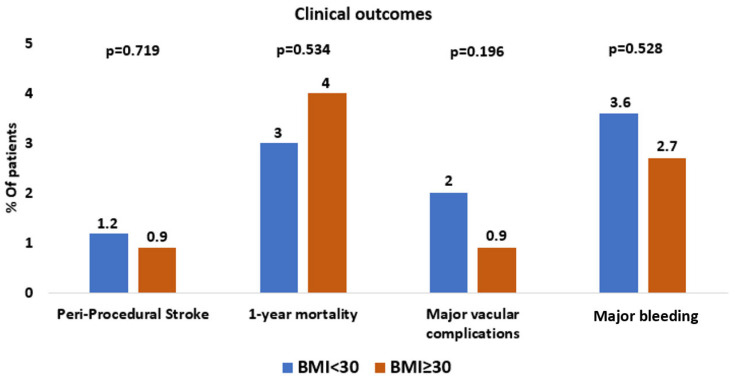
Clinical outcomes.

Data regarding procedural and clinical outcomes are detailed in Table 3 and Figure 2. In the overall population of 1045 patients undergoing TAVR, procedural and clinical outcomes were favorable, with a device success rate of 92%, a low incidence of peri-procedural stroke (1.1%), low in-hospital mortality (0.4%), and a 1-year mortality rate of 3%. Major vascular complications were observed in 2% of patients, life-threatening bleeding in 1%, and major bleeding events in 3.4%. Coronary obstruction and peri-procedural myocardial infarction were rare, occurring in only 0.2% and 0.4% of patients, respectively. When outcomes were analyzed according to BMI, device success rates remained high and comparable between obese and non-obese patients (93% vs. 92%). The incidence of peri-procedural stroke did not differ significantly between the two groups (0.9% vs. 1.2%, *p* = 0.711). The absolute risk difference for stroke between BMI groups was 0.29%, corresponding to a relative risk of 1.31 (95% CI 0.29–5.91). Similarly, there were no significant differences in in-hospital mortality (0% vs. 0.5%, *p* = 0.586) or 1-year mortality (4% vs. 3%, *p* = 0.534). Rates of major vascular complications (0.9% vs. 2%, *p* = 0.196), life-threatening bleeding (1% vs. 0.9%, *p* = 0.599), and major bleeding (2.7% vs. 3.6%, *p* = 0.528) were also comparable between obese and non-obese patients.

A subgroup analysis comparing patients with ACT values above or below 250 s revealed no significant difference in periprocedural stroke rates (1% vs. 1.5%, *p* = 0.604) (Table 4). Multivariable analysis was performed to identify independent predictors of device success (Table 5). Longer procedure duration (OR 0.981, 95% CI 0.971–0.991; *p* < 0.001) and higher contrast volume (OR 0.993, 95% CI 0.987–0.998; *p* = 0.010) were independently associated with reduced device success. In contrast, valve repositioning was associated with a higher probability of success (OR 2.382, 95% CI 1.361–4.170; *p* = 0.002).

## 5. Discussion

In this study, the overall population of 1045 patients undergoing TAVR demonstrated high procedural success and favourable short and mid-term clinical outcomes, with low rates of peri-procedural stroke, bleeding complications, vascular complications, and mortality, despite a mean intraprocedural heparin dose of 47 U/kg and a mean ACT value of 218 s. Within this overall cohort, patients were analysed according to BMI < 30 or ≥30. Despite significant differences in heparin dosing and ACT values between groups, procedural characteristics and outcomes, including device success, periprocedural stroke, bleeding, vascular complications, in hospital and 1-year mortality were comparable. A subgroup analysis based on ACT values above or below 250 s showed no significant difference in periprocedural stroke rates. Overall, TAVR demonstrated high procedural success and favourable short and mid-term outcomes regardless of BMI. Multivariable analysis identified that longer procedure duration and higher contrast volume were associated with reduced device success, whereas valve repositioning was associated with a higher likelihood of achieving success.

In contrast to coronary interventions where heparin dosing and ACT targets are well defined and supported by strong clinical evidence [8,15], anticoagulation strategies during TAVR remain largely empirical and based on expert consensus [9]. Previous studies have shown conflicting results regarding the relationship between ACT levels and clinical outcomes during TAVR, with some suggesting higher ACT targets reduce thromboembolic events while others found no clear benefit [12,16]. Considerable variability exists between centers and operators regarding heparin dose adjustment and target ACT values, particularly in obese patients [17,18]. In our study, despite significant differences in heparin dosing and ACT levels between BMI groups, procedural success and safety outcomes were comparable. These findings suggest that moderate variations in anticoagulation intensity during TAVR may not substantially impact thromboembolic or bleeding risk. One possible explanation is the distinct procedural environment, since TAVR involves manipulation within the aorta and valve annulus, which are large caliber structures with lower thrombotic potential compared to small coronary arteries. Therefore, achieving very high ACT values may be less critical in preventing thromboembolic complications during TAVR. However, given the low number of stroke events and wide confidence intervals, these results should be interpreted with caution, as clinically meaningful differences cannot be excluded. In addition, differences in baseline cerebrovascular risk between BMI groups may have influenced stroke risk and should be considered when interpreting the absence of a detected association. Accordingly, the absence of a detected association should not be interpreted as evidence that no association exists. These results reflect the experience of a single high-volume center in a selected population of patients not receiving chronic oral anticoagulation and without atrial fibrillation. As such, our findings should be considered hypothesis-generating and may inform the design of prospective studies evaluating anticoagulation strategies during TAVR

The findings of this study may have several important clinical implications. The absence of differences in procedural or safety outcomes between obese and non-obese patients despite variations in heparin dosing and ACT values suggests that anticoagulation management during TAVR may not need to be strictly weight adjusted. This supports a more individualized approach rather than a universal target strategy. This individualized approach would consider factors such as baseline coagulation profile, procedural complexity, and bleeding risk. Moreover, since higher ACT levels did not translate into improved outcomes in this study, excessive anticoagulation may not provide additional benefit and could potentially increase bleeding risk, thus increasing the need for protamine administration, which may complicate management if a covered stent becomes necessary. These results highlight the need for standardized, evidence-based anticoagulation protocols in TAVR and emphasize the importance of future prospective trials to define optimal ACT targets and heparin dosing strategies.

This study has important limitations that should be acknowledged. First, as a retrospective observational analysis, the findings may be affected by residual confounding and potential sources of bias. Second, although the study included a relatively large cohort, its single-center design may restrict the applicability of the findings to other institutions with different patient profiles, procedural techniques, and local practice patterns, including differences in practice environment, regional characteristics, and procedural duration [19]. Third, stroke events may have been under-detected, as identification relied on clinical suspicion during in hospital follow up before discharge rather than systematic neurological assessment. No routine independent neurological evaluation was performed, potentially underestimating minor or non-disabling strokes, and standardized severity scores (NIHSS or modified Rankin Scale) were not available. In addition, neurological events occurring after hospital discharge (e.g., within 30 days) were not systematically captured, which may further underestimate the true stroke incidence. Fourth, some patients from the original database were excluded due to missing data, which could introduce selection bias and slightly limit the applicability of the results. Finally, given the low number of stroke events, the study was underpowered to detect small between-group differences in stroke risk; therefore, modest differences cannot be excluded. Moreover, the imbalance in baseline stroke risk factors between BMI groups may have introduced residual confounding that could not be fully addressed due to the small number of events.

In conclusion, this study suggests that despite differences in heparin dosing and ACT values, procedural characteristics and clinical outcomes of TAVR, including peri-procedural stroke, were low in the overall population and similar between obese and non-obese patients. These results may suggest that variations in intraprocedural anticoagulation management may not significantly affect stroke risk. However, given the low number of stroke events, the absence of a detected association should not be interpreted as evidence that no association exists. Future randomized studies are warranted to define optimal heparin dosing and ACT targets and to establish standardized anticoagulation strategies during TAVR.

## 6. Clinical Perspectives

- There are no clear evidence-based guidelines defining optimal heparin dosing or target activated clotting time (ACT) values during TAVR.
- Differences in heparin dosing and ACT values did not impact clinical outcomes, including periprocedural stroke, reassuring operators that real-world anticoagulation variability during TAVR, particularly in obese patients, is unlikely to compromise safety.
- Future randomized studies are warranted to define optimal heparin dosing and ACT targets during TAVR.

## Figures and Tables

**Table 1:** Baseline characteristics.

Characteristic	Overall	BMI < 30	BMI ≥ 30	*p*-Value
n	1045	827	218	
Age, years, (mean ± SD)	82 ± 6	83 ± 6	80 ± 6	<0.001
Gender, male n (%)	583 (56)	464 (56)	119 (54)	0.688
Height, cm (mean ± SD)	165 ± 9	165 ± 8	164 ± 9	0.354
Weight, kg (mean ± SD)	73 ± 14	68 ± 11	91 ± 12	<0.001
BMI, kg/m^2^ (mean ± SD)	27 ± 4	25 ± 3	34 ± 3	<0.001
Cardiovascular Comorbidities				
Dyslipidemia, n (%)	512 (49)	400 (48)	112 (51)	0.429
Hypertension, n (%)	786 (75)	596 (72)	190 (87)	<0.001
Diabetes mellitus, n (%)	238 (23)	164 (20)	74 (34)	<0.001
Coronary artery disease, n (%)	419 (40)	337 (41)	82 (38)	0.401
Prior PCI, n (%)	293 (28)	234 (28)	59 (27)	0.719
Concomitant pre-TAVR PCI, n (%)	261 (25)	212 (25)	49 (22)	0.338
Prior CABG, n (%)	40 (4)	30 (4)	10 (4)	0.511
Prior CVA/TIA, n (%)	70 (6)	63 (8)	7 (3)	0.021
PVD, n (%)	105 (10)	84 (10)	21 (10)	0.819
Baseline ECG				
First degree AV block, n (%)	139 (13)	108 (13)	31 (14)	0.653
RBBB, n (%)	116 (11)	93 (11)	23 (10)	0.771
LBBB, n (%)	72 (7)	54 (6)	18 (8)	0.370
Baseline TTE data				
LVEF%, (mean ± SD)	61 ± 10	61 ± 10	61 ± 9	0.598
AVA, cm^2^ (mean ± SD)	0.80 ± 0.17	0.79 ± 0.17	0.82 ± 0.16	0.017
Mean Aortic gradient, mmHg, (mean ± SD)	49 ± 12	49 ± 12	50 ± 11	0.155
Surgical Risk				
Euroscore II, median (Q1 ± Q3)	2.43 (1.71 ± 3.82)	2.45 (1.74 ± 3.93)	2.32 (1.63 ± 3.54)	0.022
STS Score, median (Q1 ± Q3)	2.81 (1.89 ± 4.25)	2.87 (1.89 ± 4.33)	2.56 (1.82 ± 4.04)	0.099

BMI = Body Mass Index; PCI = Percutaneous coronary intervention; TAVR = Transcatheter aortic valve replacement; CABG = Coronary artery bypass graft; CVA = Cerebrovascular Accident; TIA = Transient ischemic attack; PVD = Peripheral vascular disease; LVEF = Left ventricle ejection fraction; AVA = Aortic valve area.

**Table 2:** Peri-procedural data.

	Overall	BMI < 30	BMI ≥ 30	*p*-Value
N	1045	827	218	
SEVs, n (%)	668 (64)	528 (64)	140 (64)	0.918
Procedure duration, min (mean ± SD)	46 ± 19	46 ± 19	46 ± 18	0.890
Fluoroscopy time, min (mean ± SD)	12 ± 6	12 ± 6	11 ± 5	0.316
Contrast medium, ml (mean ± SD)	82 ± 36	82 ± 38	80 ± 29	0.303
Heparin, U/Kg (mean ± SD)	47 ± 14	50 ± 14	40 ± 11	<0.001
ACT, s (mean ± SD)	218 ± 51	221 ± 52	208 ± 45	<0.001
Protamine use, n (%)	20 (1.9)	17 (2)	3 (1.3)	0.515
Pre-dilatation, n (%)	243 (23)	197 (24)	46 (21)	0.319
Post-dilatation, n (%)	191 (18)	147 (18)	44 (20)	0.413
Valve repositioning, n (%)	359 (34)	286 (34)	73 (33)	0.762
Need for second valve, n (%)	9 (1)	9 (1)	0 (0)	0.217

SEV = Self-expandable valves; ACT = Activated Clotting Time.

**Table 3:** Procedural and Clinical outcomes.

	Overall	BMI < 30	BMI ≥ 30	*p*-Value
n	1045	827	218	
Device success *, n (%)	962 (92)	760 (92)	202 (93)	0.711
Peri-procedural stroke, n (%)	12 (1.1)	10 (1.2)	2 (0.9)	0.719
Disabling stroke, n (%)	6 (0.5)	5 (0.6)	1 (0.4)	0.634
Non-Disabling stroke, n (%)	6 (0.5)	5 (0.6)	1 (0.4)	0.634
In hospital death, n (%)	4 (0.4)	4 (0.5)	0 (0)	0.586
1-year mortality, n (%)	36 (3)	27 (3)	9 (4)	0.534
Major vascular complication **, n (%)	21 (2)	19 (2)	2 (0.9)	0.196
Life-threatening bleeding †, n (%)	11 (1)	8 (0.9)	3 (1)	0.599
Major bleeding ‡, n (%)	36 (3.4)	30 (3.6)	6 (2.7)	0.528
Permanent Pacemaker, n (%)	135 (13)	108 (13)	27 (12)	0.792
Coronary obstruction, n (%)	3 (0.2)	3 (0.3)	0 (0)	0.495
Peri-procedural MI, n (%)	5 (0.4)	5 (0.5)	0 (0)	0.586

MI = Myocardial infarction. * Defined as the absence of procedural mortality, proper positioning of a single prosthetic valve, and a mean aortic gradient below 20 mmHg or peak velocity < 3 m/s and no moderate or severe prosthetic aortic valve regurgitation. ** Any thoracic aortic dissection OR vascular access-site injury resulting in death, the need for ≥4 units of blood transfusion, requirement for percutaneous or surgical repair, or causing irreversible end-organ damage; OR distal embolization (non-cerebral) from a vascular source that necessitates surgical treatment or leads to amputation or permanent end-organ injury. † Fatal bleeding (BARC type 5) OR bleeding involving a critical organ, including intracranial, intraspinal, intraocular, or pericardial bleeding requiring pericardiocentesis, or intramuscular bleeding associated with compartment syndrome (BARC type 3b and 3c) OR bleeding leading to hypovolemic shock or marked hypotension requiring vasopressors or surgical intervention (BARC type 3b) OR overt bleeding with a hemoglobin decrease of ≥5 g/dL or transfusion of whole blood or packed red blood cells (RBCs) ≥4 units (BARC type 3b). ‡ Overt bleeding accompanied by a hemoglobin decrease of at least 3 g/dL or requiring transfusion of 2–3 units of whole blood or RBCs.

**Table 4:** Stroke risk by ACT values.

	ACT ≤ 250 s	ACT > 250 s	*p*-Value
n	845	200	
Peri-procedural stroke, n (%)	9 (1)	3 (1.5)	0.604
Disabling stroke, n (%)	5 (0.6)	1 (0.5)	0.677
Non-Disabling stroke, n (%)	4 (0.4)	2 (1)	0.323

ACT = Activated Clotting Time.

**Table 5:** Multivariant analysis for Device success *.

	Univariable OR (95% CI)	*p*-Value	Multivariable OR (95% CI) *	*p*-Value
Age,	0.998 (95% CI, 0.963–1.035)	0.915		
Gender, male	1.016 (95% CI, 0.647–1.596)	0.944		
BMI, kg/m^2^	1.113 (95% CI, 0.631–1.962)	0.711		
Dyslipidemia	0.840 (95% CI, 0.536–1.316)	0.446		
Hypertension	0.960 (95% CI, 0.569–1.622)	0.880		
Diabetes mellitus	1.256 (95% CI, 0.714–2.212)	0.429		
Coronary artery disease	1.345 (95% CI, 0.838–2.158)	0.219		
Prior PCI	1.341 (95% CI, 0.789–2.281)	0.278		
Prior CABG	0.768 (95% CI, 0.266–2.212)	0.625		
Prior CVA/TIA	0.646 (95% CI, 0.298–1.399)	0.268		
PVD	0.911 (95% CI, 0.442–1.879)	0.802		
LVEF%	0.985 (95% CI, 0.962–1.009)	0.210		
AVA, cm^2^	0.254 (95% CI, 0.069–0.938)	0.040	0.214 (95% CI, 0.056–0.817)	0.024
Mean Aortic gradient, mmHg	0.997 (95% CI, 0.979–1.015)	0.746		
Euroscore II	0.970 (95% CI, 0.925–1.018)	0.218		
STS Score	1.045 (95% CI, 0.948–1.152)	0.375		
Valve type (BEV vs. SEV)	0.756 (95% CI, 0.480–1.193)	0.230		
Procedure duration, min	0.977 (95% CI, 0.968–0.986)	<0.001	0.981 (95% CI, 0.971–0.991)	<0.001
Contrast medium, ml	0.989 (95% CI, 0.985–0.994)	<0.001	0.993 (95% CI, 0.987–0.998)	0.010
Pre-Dilation	1.022 (95% CI, 0.600–1.743)	0.935		
Post-Dilation	0.634 (95% CI, 0.376–1.068)	0.087	0.612 (95% CI, 0.355–1.056)	0.078
Valve repositioning	1.841 (95% CI, 1.085–3.125)	0.024	2.382 (95% CI, 1.361–4.170)	0.002

PCI = Percutaneous coronary intervention; CABG = Coronary artery bypass graft; CVA = Cerebrovascular Accident; TIA = Transient ischemic attack; PVD = Peripheral vascular disease; LVEF = Left ventricle ejection fraction; AVA = Aortic valve area; BEV = Balloon-expandable valve; SEV = Self-expandable valves. * Device Success was Defined as the absence of procedural mortality, proper positioning of a single prosthetic valve, and a mean aortic gradient below 20 mmHg or peak velocity < 3 m/s and no moderate or severe prosthetic aortic valve regurgitation.

## Data Availability

The data supporting the findings of this study are available from the corresponding author upon reasonable request.

## Supplementary Materials

The following supporting information can be downloaded at: https://www.mdpi.com/article/10.3390/jcm15031201/s1, Table S1: Strobe Checklist; Table S2: Statistical test details.

## Abbreviations

TAVR	Transcatheter Aortic Valve Replacement
UFH	Unfractionated heparin
BMI	Body Mass Index
ACT	Activated clotting times
CVA	Cerebrovascular accident
